# Effects of controversial statements on social media regarding the oral glucose tolerance testing on pregnant women in Turkey

**DOI:** 10.3934/publichealth.2020003

**Published:** 2020-01-10

**Authors:** Gökmen Özceylan, Dilek Toprak

**Affiliations:** 1 Family Medicine Specialist, Çorlu Reşadiye Family Medicine CenterOrchid, Tekirdağ, Turkey; 2 Namık Kemal University, Medical School head of family medicine department, Tekirdağ, Turkey

**Keywords:** oral glucose tolerance test, social media, pregnancy, gestational diabetes, screening test

## Abstract

Discussions of the use of the oral glucose tolerance test (OGTT) took off when a Turkish scientist claimed in the media that “the OGTT is poisoning babies” in 2014. The aim of present study investigates the effects of controversies in the media and on the Internet on the attitudes and behaviors of women in regards to the OGTT. The research was designed as a descriptive, cross-sectional study. The universe of the study was women aged 18–45 years in Turkey. Included in the study were 358 women of childbearing age who attended family health center outpatient clinics in January 2019. A questionnaire was administered during face-to-face interviews to those who provided consent for participation in the study. The data was analyzed using SPSS 22.0 software. Chi-square test was used to compare the between-group qualitative data. The results were evaluated based on an alpha value of 0.05. Results: Of the participating women, 18.99% (n = 88) were unaware of the OGTT. Of participant, 41.89 (n = 151) delivered “Iwill take OGTT in the future”. 27.09% (n = 97) delivered “I will not” and 11.73% (n = 42) were hesitant. Of the participants, 67.32% (n = 241) reported having been pregnant in the past. Of the participant, 62.24% (n = 150) delivered that they had OGTT in the past. The reasons given for not undergoing the OGTT in past pregnancies were 29.45% (n = 38) unaware during pregnancy, 28.68% (n = 37) delivered “my family physician did not recommend it”. But the ones who delivered that in the future will not take OGTT, their reasons were 56.66% (n = 34) delivered “heard from media and internet sources” that the test was harmful. The results of the study indicate that public trust of OGTT and taking OGTT rates are declining in Turkey. While women can obtain beneficial information from media and Internet sources, misinformation can easily shake their confidence in any scientific data.

## 1. Introduction

Gestational diabetes (GDM) refers to diabetes diagnosed for the first time during pregnancy, which is known harm the mother and baby, and that resolves after the termination of the pregnancy [1],[2]. Complications with diabetes are more commonly seen in expectant mothers diagnosed with GDM, which can affect also the fetus. Treatment reduces the frequency of these complications, indicating the importance of early diagnosis. The high prevalence of the risk factors raises the question of whether screening of the general population or high-risk pregnant women should be carried out [3], and a wide range of tests have been developed and implemented to date for the diagnosis of and screening for GDM.

Discussions of the use of the oral glucose tolerance test (OGTT) took off when a Turkish scientist claimed in the media that “the OGTT is poisoning babies” [4]. The Turkish Society of Obstetrics and Gynecology asserted the contrary, and the Turkish Ministry of Health declared that “this scientist's statement does not reflect reality; this test is highly recommended during pregnancy” [5],[6]. Despite these counter statements, a lack of public trust in the OGTT started to emerge. The present study investigates the effects of controversies in the media and on the Internet on the attitudes and behaviors of women in regards to the OGTT.

## 2. Materials and methods

The research was designed as a descriptive, cross-sectional study. The universe of the study was women aged 18–45 years. Included in the study were 358 women of childbearing age who attended family health center outpatient clinics in January 2019. A questionnaire was administered during face-to-face interviews to those who provided consent for participation in the study. The first section of the questionnaire investigated the demographic data of the women, their pregnancy history and whether they had undergone the OGTT in previous pregnancies. The second section examines whether they would undergo the OGTT in the future if they become pregnant. Women who hesitated in providing an answer, or who stated that they would be against undergoing an OGTT test were asked for the specific reasons behind their decision. The answers were then grouped and analyzed.

Women aged 18–30 years were included in the “young women” group and those aged 31–45 years were assigned to the “older women group”. Illiterate women those who graduated from primary or secondary were classified as having a low educational level, while highschool or university graduates were classified as having a high educational level. The monthly minimum wage was taken as the basis for the monthly income classification, with a salary of 2050 TL or lower considered low income, 2051–4100 TL considered moderate income, and 4101 TL considered high income.

The data was analyzed using SPSS 22.0 software. A side from the descriptive statistics (mean, standard deviation, percentage, maximum, minimum), a Chi-square test was used to compare the between-group qualitative data. The results were evaluated based on an alpha value of 0.05.

Ethics approval and consent to participate: Date 08.01.2019 and 13177 number of ethic approvel by Tekirdağ Provincial Health Directorate. Consent for publication: Date 13.01.2019 and 13179 number of Tekirdağ Provincial Health Directorate. We obtained from the all study participants written consent. This written consent was approved by the ethical approval by Tekirdağ Provincial Health Directorate.

Availability of data and material: The datasets used and/or analysed during the current study are available from the corresponding author on reasonable request.

## 3. Results

The mean age of the participants was 30.97 ± 7.03 (min = 18, max = 45). The demographic characteristics of the participants are summarized in Table 1.

**Table 1.** Socio-demographic characteristics of the participants.

Participants	n (%)
Age	
18–30	204 (56.98)
31 years and older	154 (43.02)
Marital status	
Married	269 (75.14)
Single	89 (24.86)
Monthly income level	
Low	111 (31.01)
Moderate	161 (44.97)
High	86 (24.02)
Educational Level	
Illiterate	13 (3.63)
Primary School	101 (28.21)
Secondary School	41 (11.45)
High-School	90 (25.14)
University	113 (31.57)

Of the participating women, 18.99% (n = 88) were unaware of the OGTT. Awareness of the test was lower in single women than in married women, and lower in women with a low-income level than in those with a high-income level (p < 0.00; p = 0.001). The level of awareness was significantly higher among those with a history of pregnancy than among those who had never been pregnant (p < 0.001).

Of the participants, 67.32% (n = 241) reported having been pregnant in the past. A comparison of the OGTT history of those women and the views of all the participating women about the possibility of taking the OGTT in the future is made in Table 2.

**Table 2.** OGTT behaviors of women who have been pregnant in the past compared with the views of all participants about undergoing the OGTT in the future.

Past pregnancy (n = 241)	% (n)	Prospective pregnancy(n = 358)	% (n)
I took the OGTT	62.24 (n = 150)	I will take the OGTT	41.89 (n = 151)
I did not take the OGTT	37.76 (n = 91)	I will not take the OGTT	27.09 (n = 97)
		Hesitant	11.73 (n = 42)
		I am unaware of the test	18.99 (n = 68)

**Figure 1.**
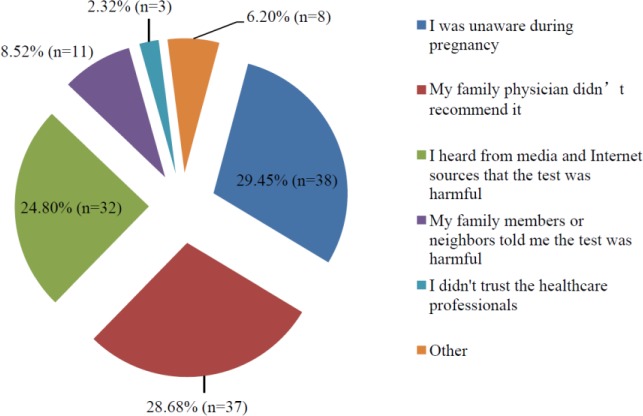
Distribution of reasons for not having undergone the OGTT in past pregnancies (n = 91).

The rate of not having undergone the OGTT in past pregnancies was higher among older women and in those with lower levels of educational and income (p < 0.001; p < 0.001; p = 0.005). The reasons given for not undergoing the OGTT in past pregnancies are presented in Figure 1.

Women who were unaware of the OGTT in past pregnancies, and those who did not undergo the test for this reason, were older and had a lower level of education and income (p < 0.001; p < 0.001; p = 0.009).

Those who stated that they had not undergone the OGTT after being influenced by news in the media and on the Internet were younger, and had a higher level of both education and income (p = 0.001; p = 0.002; p = 0.004).

**Figure 2.**
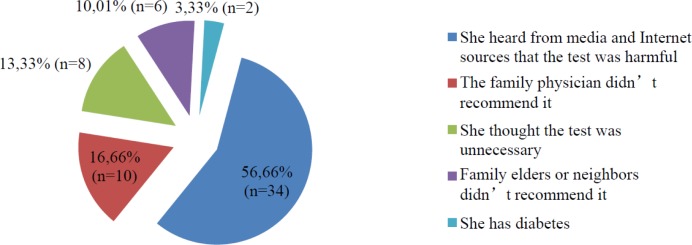
Distribution of reasons reported by 60 women who expressed hesitation (n = 21) or who were against (n = 39) OGTT, although they had undergone the OGTT in past pregnancies.

Among the 150 women who had undergone an OGTT in a past pregnancy, 60.00% (n = 90) reported that they would undergo an OGTT again if they become pregnant in the future, and had been advised to do so by the family physician, whereas 14.00% (n = 21) were hesitant and 26.00% (n = 39) reported that they would not undergo the test. The distribution of the reasons given by 60 women who stated being hesitant or against taking the test is presented in Figure 2.

**Table 3.** Analysis of responses to the question of “Would you take the OGTT if you become pregnant in the future?” according to demographic data.

	Would you take the OGTT if you become pregnant in the future?				
	No %(n)	Yes %(n)	Hesitant %(n)	I am unaware %(n)	p
Age distribution					0.035
18–30	30.4 (62)	34.8 (71)	12.3 (25)	22.5 (46)	
31 years and older	27.4 (35)	48.8 (80)	10.4 (17)	13.4 (22)	
Educational level					> 0.05
Low	26.3 (41)	42.9 (67)	12.8 (20)	17.9 (28)	
High	27.7 (56)	41.6 (84)	10.9 (22)	19.8 (40)	
Marital status					< 0.001
Married	29.4 (79)	48.3 (130)	13.4 (36)	8.9 (24)	
Single	20.2 (18)	23.6 (21)	6.7 (6)	49.4 (44)	
Monthly income level					0.002
Low	27.9 (31)	33.3 (37)	8.1 (9)	30.6 (34)	
Moderate	23.0 (37)	46.0 (74)	15.5 (25)	15.5 (25)	
High	33.7 (29)	46.5 (40)	9.3 (8)	10.5 (9)	

**Table 4.** Analysis of reasons reported by the 139 women who reported being against the OGTT in the future, or who expressed hesitation, according to age.

	18–30 years %(n)	31–45 years %(n)	Total %(n)	p
I learned from Internet sources/media that the test is harmful to the baby	45.32 (63)	28.77 (40)	74.09 (103)	0.001
I don't think it is necessary	8.63 (12)	2.15 (3)	10.78 (15)	> 0.05
My family physician does not recommend it	2.15 (3)	4.31 (6)	6.46 (9)	> 0.05
My family does not recommend it	0.72 (1)	2.15 (3)	2.87 (4)	> 0.05
I have diabetes	0.72 (1)	2.15 (3)	2.87 (4)	> 0.05
I took the test during my first pregnancy; it was very difficult	1.44 (2)	0.72 (1)	2.16 (3)	> 0.05
Other	0 (0)	0.75 (1)	0.75 (1)	> 0.05

**Table 5.** Number of expectant mothers presenting to the study center between 2014 and 2018, and the yearly distribution of the OGTTs performed on these women.

Year	Number of expectant mothers	Number of OGTTs	%
2014	245	100	40.81
2015	650	171	26.30
2016	698	119	15.90
2017	543	100	18.41
2018	579	71	12.26

Analysis of attitudes towards the OGTT in prospective pregnancies based on demographic data is presented in Table 3.

The age distribution of the 139 participating women who reported being against the OGTT in the future, or who expressed hesitation, is presented in Table 4.

The data in Table 5 was garnered from expectant mothers who attended the family health center between 2014 and 2018,with the number of OGTTs performed on these women being evaluated retrospectively.

## 4. Discussion

The prevalence of diabetes mellitus is increasing worldwide in parallel to changes in lifestyles and the increasing prevalence of obesity. This has led to an increase in the use of diagnostic and screening tests [7]. There have been a number of studies suggesting increased oxidative stress in fetuses associated with OGTT screening in literature, and these studies have provoked discussions of the safety of the test [8]. A Turkish scientist started a new discussion in 2014 when she stated that “the OGTT is poisoning fetuses”. This controversial statement led to an increase in the perception that “the OGTT is harmful”, and increased hesitance and distrust in the test.

Literature reports the level of awareness of the OGTT to be around 80% in Croatia, 83% in Spain [9],[10], 39% in Pakistan and 39.6% in Qatar [11],[12]. Although no such study has been encountered in Turkey reporting on the level of awareness of the OGTT, the present study reports that approximately eight out of 10 women are aware of the test. Among the participating women, 30% reported being unaware of the test in their past pregnancies, giving this as the reason why they had not undergone the test, and approximately 18% were currently unaware of the test. This data suggests that controversies in the media and the increasing prevalence of diabetes over time have increased the level of awareness, although two out of 10 women are still unaware of the test. Those who were unaware of the test in the present study were mostly single, were in the young age group and had a low level of income. Accordingly, the authors believe that marriage and preconception training programs targeting young and single women should be implemented in order to increase public awareness of the OGTT.

It has been reported that 63% of women in the United States underwent screening for gestational diabetes [13]. A cohort study conducted in two large centers in Europe revealed that 67% of women had undergone screening for GDM using the OGTT [14]. A study conducted in an Ankara Training and Research Hospital in 2012 based on hospital data reported that 7.5% of patients presenting to the hospital underwent the OGTT [15]. Consistent with literature, the rate of women undertaking the OGTT during pregnancy was 62% in the present study, although it was found that the rate of those considering undergoing the OGTT when they become pregnant in the future was only 42%. Furthermore, one in 10 women were hesitant, and around three out of 10 women said that they would not consider undergoing the OGTT. An analysis of the records of the health center in which the present study was conducted revealed that 40% of the women presenting to the center in 2014 undertook the OGTT, but that this rate was only 12% in 2018, indicating a significant decrease in the rate of people undergoing the OGTT and in those intending toundergo the test in the future. Although there has been an increase in the awareness of the OGTT, there has been a decrease in those taking the test, indicating an emerging distrust in the safety of the test. We consider therefore, that healthcare professionals should carry out more comprehensive studies into the safety of this test and share their results with the public through appropriate channels of communicationand using appropriate language.

An analysis of the reasons given for not undergoing screening with the OGTT revealed many studies reporting a direct relationship with difficulties in accessing healthcare providers, cultural and ethnic differences, age, gender, and education and income levels [11],[12],[16]–[18]. The most common reason for not undergoing the OGTT in past pregnancies was reported to be unawareness of the test, followed, in descending order, by not being advised by the attending doctor monitoring the course of the pregnancy, and reports in the media and from Internet sources that the test is harmful, at similar rates of around 30%. When the same participants were asked about taking the OGTT in the future, six out of the 10 women who were hesitant or expressed a reluctance to undergo the OGTT reported having learned from Internet sources that the test is harmful. Accordingly, a significant proportion of the women who undertook the OGTT in past pregnancies had been negatively affected by the controversy, and had either developed a hesitancy to undergo the test or had decided outright not to undergo the test.

Approximately one-fifth of the women who participated in the study stated that they would not have OGTT and that family physicians did not recommend the test. This result shows that family physicians may have been negatively affected by these discussions. Pregnant follow-up of family physicians in a very important place in Turkey about the benefits of this test is subjected to re-education program, if any doubt, it is necessary to remove these doubts.

Of the 10 women who stated that they were against or reluctant to undergo the OGTT, seven stated that negative media and Internet sources had influenced their decision. The fact that most of these participants were young women suggests that the rate of women undergoing this test will continue to decrease in the future. For this reason, measures must be taken to prevent the spread of disinformation and misdirection, and to restore trust in this screening test, particularly among young single women. In this regard, it is recommended that education projects be developed.

## 5. Conclusion

Scientists working in the field of medicine should consider the long-term consequences of their media statements. Statements that are not supported by scientific data put public health at risk. We recommend testing the accuracy of their statement based on scientific data, and carefully weigh up the possible effects of their statement on public health.

The results of the present study indicate that while women can obtain beneficial information women from media and Internet sources, misinformation can easily shake their confidence in any scientific data. Health policy-makers are recommended to regulate any statements made in the media or on the Internet related to public health through laws and legislation.

In particular, local administrators can have a significant effect on eliminating the distrust of this test through training and information projects. Local health managers should ensure that family physicians pay attention to the distrust developed against this test by issuing the necessary formal warnings to family medicine units. In order to regain confidence in the test, Family physicians, should be interviewed with pregnant women about the benefical of the test, during follow-up.

Furthermore, media organizations are advised to consult a committee or a council of the relevant profession and verify the accuracy of the relayed information before broadcasting such statements.
